# Associations between glycan signature alterations and the cellular antigenic properties of passaged chondrocytes

**DOI:** 10.3389/fimmu.2024.1475473

**Published:** 2024-11-25

**Authors:** Kentaro Homan, Taiki Tokuhiro, Tomohiro Onodera, Hisatoshi Hanamatsu, Jun-ichi Furukawa, Taku Ebata, Masatake Matsuoka, Ken Kadoya, M. Alaa Terkawi, Norimasa Iwasaki

**Affiliations:** ^1^ Department of Orthopaedic Surgery, Faculty of Medicine and Graduate School of Medicine, Hokkaido University, Sapporo, Japan; ^2^ Department of Advanced Medicine for Locomotor System, Faculty of Medicine and Graduate School of Medicine, Hokkaido University, Sapporo, Japan; ^3^ Department of Biomaterial Function Regeneration Field, Faculty of Medicine and Graduate School of Medicine, Hokkaido University, Hokkaido University, Sapporo, Japan; ^4^ Institute for Glyco-core Research (iGCORE), Nagoya University, Nagoya, Japan

**Keywords:** passage culture, glycome analysis, antigenicity, chondrocyte, macrophage, cellular transplantation

## Abstract

**Background:**

Cartilage repair is a significant clinical challenge because of the limited intrinsic healing capacity. Current therapeutic strategies, such as cell transplantation therapy, aim to overcome this challenge by replacing damaged tissue with healthy cells. However, the long-term survival and functionality of transplanted cells remain major hurdles.

**Objective:**

This study investigated the impact of chondrocyte passaging on glycan profiles and their antigenic properties. We hypothesized that alterations in glycan composition due to passaging may contribute to the enhanced ability to activate macrophages, thereby affecting the outcome of cell transplantation therapy.

**Methods:**

Peritoneal macrophages and primary articular chondrocytes were isolated from C57BL/6 mice to establish direct and indirect coculture models. Macrophage activation was assessed by measuring the concentrations of IL-6 and nitric oxide in the culture supernatants or their gene expression. Glycome analysis of various glycoconjugates was performed by glycoblotting methods combined with the SALSA procedure for N-glycans and GSLs and the BEP method for O-glycans.

**Results:**

Our results revealed that direct coculture of macrophages with passaged chondrocytes increased the production of proinflammatory cytokines, including IL-6 and NO, as the number of passages increased. With increasing passage number, the expression of GD3 substantially decreased, and the expression of GM3, especially GD1a, significantly increased. Coculturing passaged GM3S knockout chondrocytes with macrophages significantly suppressed IL-6 expression, implying reduced macrophage activation.

**Conclusion:**

The observed activation of macrophages due to alterations in the glycan profile of chondrocytes provides a possible explanation for the antigenicity and immune rejection of transplanted cells.

## 1 Introduction

Cartilage repair poses a significant clinical challenge worldwide because of the limited intrinsic healing capacity of these tissues (1). Current therapeutic approaches, including cell transplantation therapy, aim to address this challenge by replacing damaged tissue with healthy cells (2–4). However, the long-term survival and functionality of transplanted cells remain major obstacles to the success of such therapies (5–8).

Previous research has highlighted the importance of cell-surface glycans in mediating cellular communication and signaling (9–11). Glycans, intricate carbohydrate structures attached to cell-surface proteins, regulate various cellular processes by facilitating or hindering the binding of ligands to their receptors (12, 13). In particular, glycans play crucial roles in modulating immune responses (14–16), with implications for tissue regeneration and repair (17).

One critical aspect of cell transplantation therapy that has received less attention is the process of passaging, wherein cells are expanded in culture to generate sufficient numbers for transplantation (18, 19). Passaging is a routine practice in cell culture, but its effects on the glycan profile and subsequent immune responses of recipients remain poorly understood. Recent evidence suggests that passaging-induced alterations in the glycan profile of chondrocytes may impact their antigenicity and immune recognition. For example, studies have shown that the induction of chondrocyte differentiation changes the glycan composition of the cell as a reflection of the differentiation process (20–22). These changes in glycan composition may affect the interaction between transplanted cells and host immune cells, such as macrophages, potentially influencing the long-term survival and functionality of the transplanted cells.

This study aims to address this gap in knowledge by investigating how the expansion of primary chondrocytes influences their antigenicity against macrophages through changes in the glycan profile. We hypothesized that passaging-induced alterations in the glycan profile of chondrocytes may affect their recognition and subsequent immune response by macrophages.

## 2 Materials and methods

### 2.1 Experimental animals

All animal experiments were performed in accordance with protocols approved by the Institute of Animal Care and Use Committee of the Hokkaido University Graduate School of Medicine. C57BL/6 mice were obtained from Japan SLC, Inc. (Shizuoka, Japan) and used as wild-type (WT) mice. GM3S null mice were generated as previously described (23). This type of null mouse was backcrossed with C57BL/6 mice for >11 generations. The GM3S genotypes of the mice were analyzed via PCR as described previously (23).

### 2.2 Isolation of mouse peritoneal macrophages

Peritoneal macrophages were isolated from six- to eight-week-old C57BL/6 mice (24). Briefly, one milliliter of 4% Brewer thioglycolate medium was injected into the peritoneal cavity, allowing the inflammatory response to proceed for four days. Then, the cells were harvested by injecting 10 mL of ice-cold PBS into this cavity and collecting the fluid from the peritoneum. Thereafter, the cells were washed three times and freshly used in further experiments.

### 2.3 Isolation of mouse primary chondrocytes and cell culture

Primary articular chondrocytes were isolated from five-day-old C57BL/6 N mice via collagenase D according to a standard protocol (22, 25). In brief, we isolated cartilage from the femoral condyles and tibial plateau. We took care to remove the hypertrophic zone from the collected tissues. Then, we treated the tissues with collagenase D in DMEM at 37°C overnight. The isolated cells were seeded into dishes or flasks. Primary chondrocytes were cultured in DMEM supplemented with 10% heat-inactivated fetal bovine serum (FBS; Sigma−Aldrich), 2 mM L-glutamine, and 25 mg/L penicillin/streptomycin. The cells were used between passages 0 and 3.

### 2.4 Direct coculture of chondrocytes with macrophages

Murine peritoneal macrophages were seeded on 24-well plates or 48-well plates at a ratio of 2×10^5^. After three hours, the medium was changed to remove the nonadherent cells, and the cells were incubated overnight at 37°C and 5% CO_2_. Mouse cultured cells were prepared as described above and resuspended at 4.0×10^5^ cells/ml. Then, 500 µl of the cell suspension was placed directly upon the monolayer of the macrophages and incubated at 37°C for 48 hours. Thereafter, the cells and supernatants were harvested for further analyses. All experiments were repeated at least two times to obtain reproducible data.

### 2.5 Indirect coculture of chondrocytes with macrophages and cultured cells

Murine macrophages and cultured cells were prepared in the same manner as those used for direct coculture. The cultured cells were seeded (2 × 10^5^ cells) onto transwell insert cell cultures (Falcon cell culture inserts, BD, Franklin Lakes, NJ, USA) and placed on a 24-well plate of macrophage monolayer cultures. After 48 hours of incubation, the cells and supernatants were harvested for further analyses.

### 2.6 Detection of interleukin-6 (IL-6) by ELISA

The concentrations of IL-6 in the culture supernatants were measured via mouse IL-6 DuoSet ELISA (R&D Systems, Inc., USA) according to the manufacturer’s instructions.

### 2.7 Quantification of nitric oxide (NO)

NO in the culture supernatant was quantified on the basis of the amount of nitrite, the product generated upon degradation of NO (26). A Griess reagent system was used for this assay according to the manufacturer’s recommendations (Promega, Tokyo, Japan).

### 2.8 Immunofluorescence (IF) of chondrocytes with macrophages

Isolated murine macrophages and primary or passaged chondrocytes were seeded onto a 15 mm micro cover glass (MASATANI, Japan) and cultured in a 24-well plate for six hours. The cells were fixed with 10% formalin (Wako, Japan), treated with 0.1% Triton X-100 for 3 min, and then incubated with 5% FBS (Sigma)-PBS (blocking buffer) for one hour at RT. The cells were incubated with primary antibodies against murine F4/80 and IL-6 (BioLegend) at 1:200 for one hour at 37°C. The bound antibody was detected by a specific secondary antibody conjugated with Alexa Fluor®594 (Jackson ImmunoResearch, West Grove, PA, USA). The cell nuclei were stained with DAPI (Dojindo Molecular Technologies, Kumamoto, Japan). Imaging was performed by using a Keyence all-in-one microscope (Itasca, IL 60143, USA).

### 2.9 RNA isolation and quantitative real-time polymerase chain reaction (PCR)

Total RNA was extracted from each sample via TRIzol reagent (Thermo Fisher Scientific). For the synthesis of cDNA, reverse transcription was performed via the GoScript TM reverse transcriptase kit (Pro-mega, Madison, USA). The cDNA samples were subjected to quantitative real-time PCR via TB Green^®^ Premix Ex Taq™ II (Takara, Japan) on a Thermal Cycler Dice Real-Time System II (model TP900; Takara Bio, Shiga, Japan) with gene-specific primers (**Supplementary Table 1**). The relative expression of each targeted gene to the expression of GAPDH was calculated via the 2^-ΔΔCt^ method.

### 2.10 Glycome analysis of various glycoconjugates

Cultured chondrocytes (> 1 × 10^6^ cells) were washed five times with PBS and collected via a scraper (Sumilon, Japan). The collected cells were resuspended in 100 mL of PBS and homogenized via an ultrasonic homogenizer (TAITEC, Saitama, Japan). The cell lysates were precipitated with EtOH, and subsequently, the proteinous pellet and supernatant fractions were separated via centrifugation (22, 27, 28). The resulting pellet was dissolved in H_2_O, and the cellular protein concentration was measured via a BCA protein assay kit (Thermo Fisher Scientific). The pellet fractions corresponding to 25 μg and 50 μg of protein were used for N-glycan and O-glycan analyses, respectively. The supernatant fraction corresponding to 100 μg of protein was concentrated for GSL analysis. Glycomic analyses of N-glycans and GSLs were performed by glycoblotting methods combined with the SALSA procedure (29, 30). O-glycome analysis was performed via β-elimination in the presence of pyrazolone analogs (BEP) as previously described (31). This methodology allows a comparative analysis of glycomes.

### 2.11 Statistical analysis

The data were statistically analyzed via an unpaired two-tailed Student’s t test and one-way analysis of variance followed by the Kruskal−Wallis test followed by Dunn’s multiple comparison test via GraphPad software (GraphPad Software, La Jolla, CA, USA). Differences between groups with a *P* value less than 0.05 were considered statistically significant. The results are presented as the means ± standard deviations (SDs).

## 3 Results

### 3.1 Immunological behaviors of macrophages to the passaged chondrocytes

**Figure 1:**
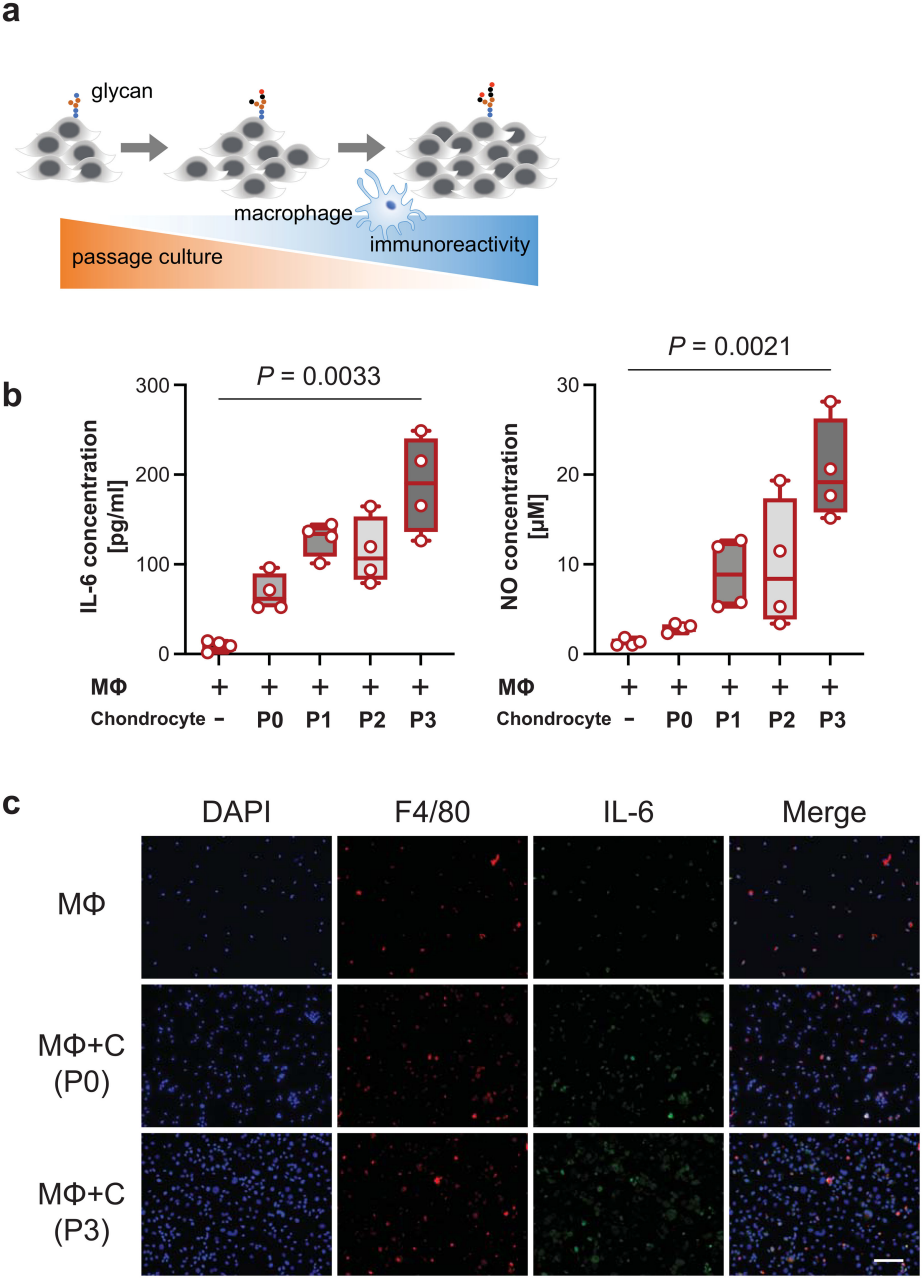
Immunological behavior through coculture with macrophages and passaged cells. **(A)**, Schematic overview of the increase in the macrophage immune response due to glycosylation with increasing passage number. **(B)**, IL-6 levels (left panel) and nitric oxide (NO) levels (right panel) released into the medium at different passage numbers of chondrocytes in a macrophage coculture system (n = 4 mice). The data are presented as the means ± s.d.s; Kruskal−Wallis test. **(C)**, IL-6-stained cells were mainly costained with F4/80-stained cells. Scale bars, 50 μm. P, passage; MΦ, macrophage; C, chondrocyte.

Direct coculture of macrophages with passaged chondrocytes increased the production of proinflammatory cytokines, including IL-6 and NO, as the number of passages increased (**Figures 1A, B**). However, indirect coculture did not activate macrophages (**Supplementary Figure 1**). NO levels were below the datable limits of the kit used. In cocultured macrophages with passaged chondrocytes, IL-6 was predominantly detected in F4/80 macrophages (**Figure 1C**).

### 3.2 Comprehensive and comparative glycomic profiling of passaged chondrocytes

#### 3.2.1 N-linked glycosylation

**Figure 2:**
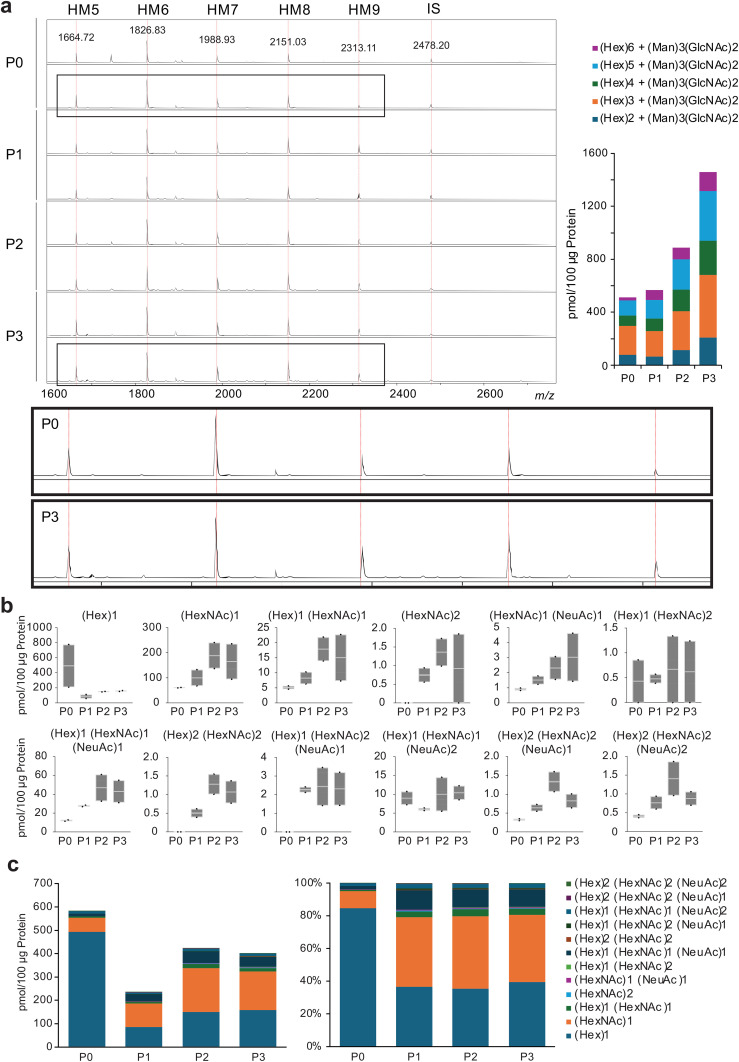
Fluctuations in N- and O-glycans with passaging. **(A)**, Spectra of chondrocytes for each passage, annotated with confirmed N-glycan structures from MALDI-TOF MS. HM, high mannose; IS, internal standard. **(B)**, Profiling of O-glycans. The glycan population detected included core substituents, and a trend toward the biosynthesis of branched O-glycans was observed. **(C)**, Absolute value and relative levels of O-glycans in chondrocytes at each passage. P, passaged.

N-glycans can be structurally classified into pauci-mannose (PM; Man1−4GlcNAc2Fuc0−1), high-mannose (HM; Man5−9GlcNAc2), and hybrid- and complex-types. Among these four types, the most distinct expression was HM-type N-glycans, which accounted for 70–80% of the total number of glycans in the passaged cells (**Figure 2A**) (**Supplementary Table 2**).

#### 3.2.2 O-linked glycosylation

For O-glycans, only in the succeeding cells were (P1-3), (HexNAc)2, (Hex)2(HexNAc)2, and (Hex)1(HexNAc)2(NeuAc)1 glycans expressed, indicating the generation of core 2 glycans (**Figure 2B**). The total amount of O-glycans decreased in passaged cells, and the expression of core 2 glycans accounted for only a small fraction of the total O-glycans in the passaged cells (**Figure 2C**) (**Supplementary Table 3**).

#### 3.2.3 Lipid-bound glycans

**Figure 3:**
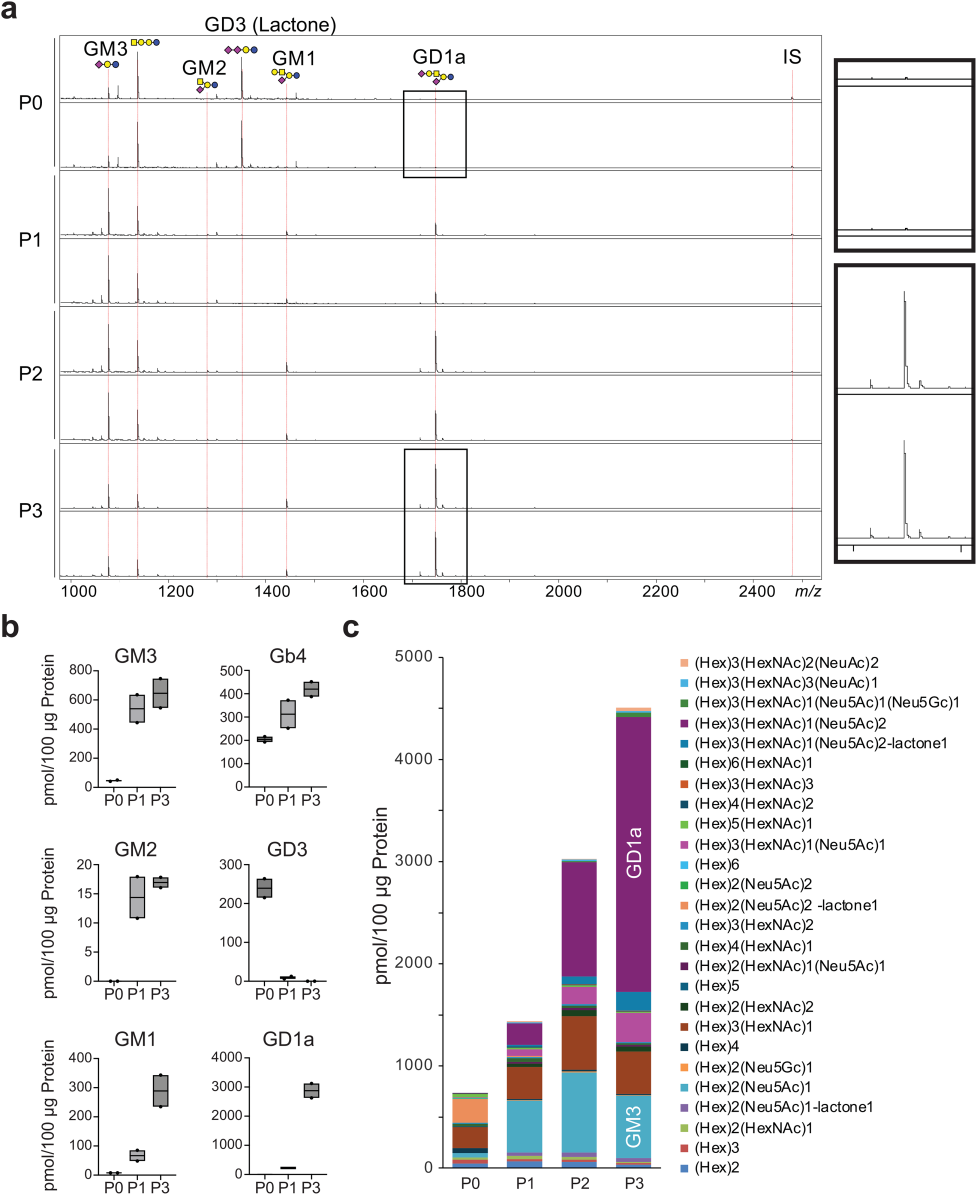
Evaluation of GSL-glycan profiles on chondrocyte per passage. **(A)**, MALDI-TOF MS spectra showing GSL-glycans on chondrocytes after successive passages. IS, internal standard. **(B)**, Expression profiles of ganglioside depending on passage number. **(C)**, The total amount of GSL-glycans increased with each successive passage. SphinGOMAP (http://www.sphingomap.org/) online databases were used for the structural estimation of the GSL glycans. NS, not significant; P, passage.

Glycosphingolipids (GSLs) are characterized by the initial addition of glucose or galactose to a ceramide unit to produce glucosylceramide (GlcCer) or galactosylceramide (GalCer), respectively. The GSL profile dynamically changed during serial passaging of the chondrocytes (**Figure 3A**). With increasing passages, GD3 expression was extremely low, and the expression of GM3, especially GD1a, predominantly increased (**Figure 3B**). GSL-glycans were markedly more abundant, as were GM3 and GD1a (**Figure 3C**). GD3 was predominantly detected in lactone form (97.0%) (**Supplementary Table 4**). This is presumed to be due to the tandem-linked α2,8-sialic acids readily forming lactones (32). In contrast, GM3 was predominantly detected as a methyl-esterified form of sialic acid (94.8%). Additionally, GD1 was detected in almost all the methyl-esterified forms (93.6%). These results suggested that GD1 is likely a structural isomer, such as GD1a, which lacks an α2,8-linked-disialyl moiety.

### 3.3 Suppression of inflammatory responses in GM3 synthase-null mice

**Figure 4:**
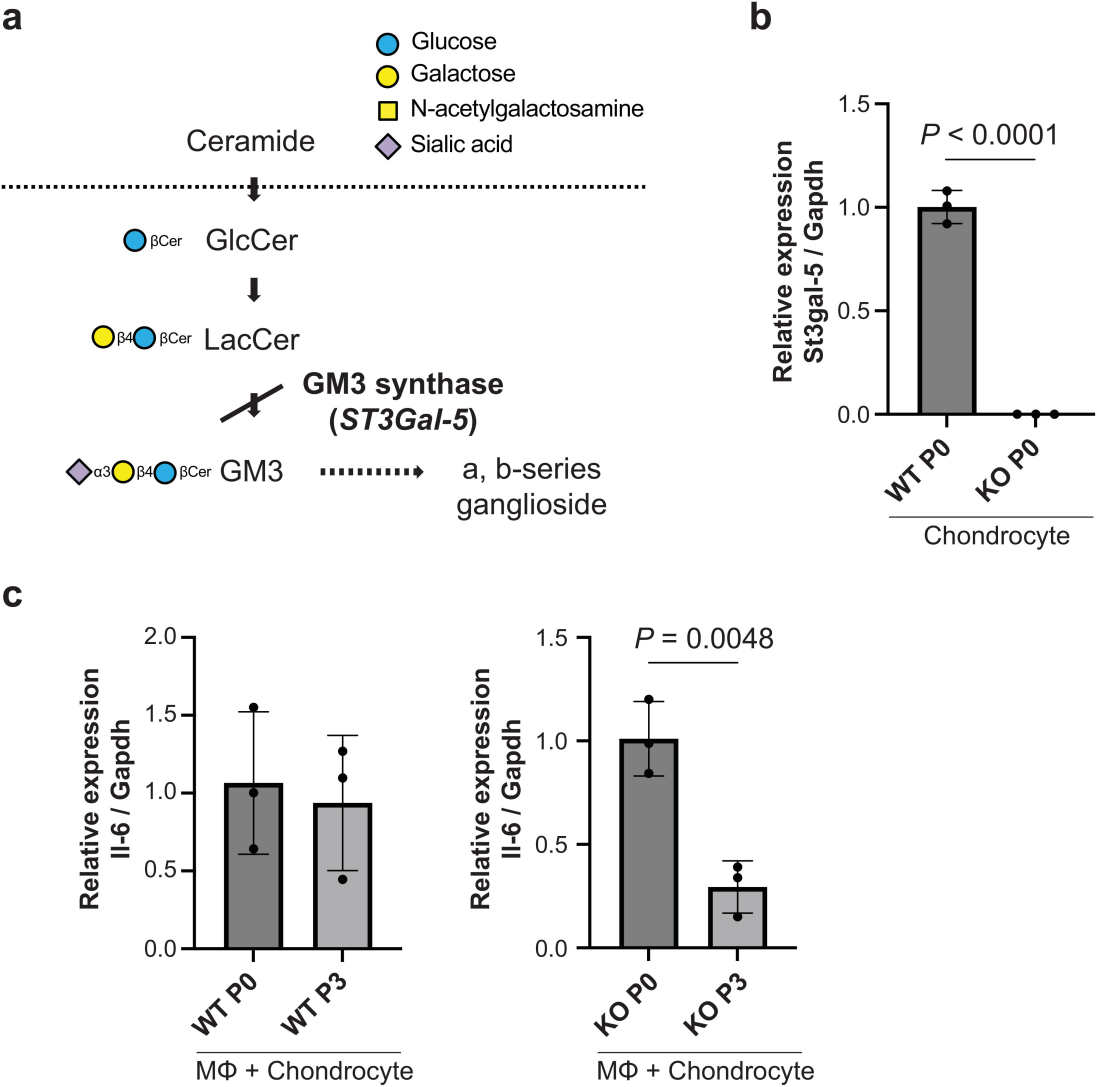
Immunological behavior through coculture with macrophages and passaged cells. **(A)**, Pathway of ganglioside synthesis showing the block in GM3 synthase (GM3S) null mice. The approved gene name for GM3S is *ST3Gal-5*. **(B)**, mRNA expression of *ST3Gal-5* in wild-type and GM3S-/- chondrocytes on passage 0 (n = 3 mice). Data are mean ± s.d.; unpaired t-test. **(C)**, mRNA expression of *il-6* in co-cultured with GM3S-/- chondrocytes and macrophage on passage 3 (n = 3 mice). Data are mean ± s.d.; unpaired t-test. P, passage; MΦ, macrophage; KO, knockout.

To determine whether gangliosides influence immunological behavior cocultured macrophages with passaged chondrocytes, we established mice with a disrupted gene (*ST3Gal-5*) encoding GM3 synthase (GM3S) (23), an a2,3 sialyltransferase that transfers a sialic acid residue to lactosylceramide to yield GM3 ganglioside (**Figure 4A**). We performed genotyping PCR using Yamashita’s primer sets (**Supplementary Figure 2**). The expression level of the GM3S genes was assessed at the mRNA level in primary chondrocytes via qRT−PCR, which revealed efficient knockout of the target *ST3Gal-4* gene in the cells (**Figure 4B**). Coculturing passaged GM3S knockout chondrocytes with macrophages significantly suppressed IL-6 expression (**Figure 4C**), indicating the reduction of macrophages activation.

## 4 Discussion

The results of this study provide evidence of the impact of chondrocyte passaging on glycan profiles and their antigenic properties. Our results demonstrated that alterations in glycan composition, particularly the increased activity of the GM3 to GD1a pathway, may contribute to the increased ability to activate macrophages. In fact, coculture experiments using chondrocytes isolated from GM3S-/- deficient mice lacking the gene encoding glycosyltransferase GM3 synthase demonstrated a reduction in the production of IL-6, a marker used for macrophage activation. These results suggest a critical role for specific glycan structures of passaged chondrocytes in modulating the macrophage response in the recipients.

The sialylation of proteins is known to be important in regulating antibody activity (15, 16), but gangliosides, GSLs containing sialic acid, have also been shown to function in immune responses. Modifications to ganglioside GM3 modulate the innate immune function of macrophages and act as pro- and anti-inflammatory endogenous Toll-like receptor 4 (TLR4) modulators (33, 34). GD1a increases IL-6 production by monocytes, and monocytes treated with GD1a promote Ig production by B cells (35). On the other hand, GM1 has a tissue-protective effect by inhibiting oxidative stress and preventing apoptosis (36–39). In the present study, the number of GM3 genes increased, and the number of downstream GD1a genes increased sharply in successional chondrocytes, suggesting that the a-series ganglioside biosynthetic pathway was activated. Our findings are consistent with those of previous studies that demonstrated the immunomodulatory effects of glycans on macrophage activation. However, this study uniquely focused on the impact of passaging-induced glycan alterations on immune reactivity, providing a more comprehensive understanding of the relationship between cell culture practices and immune responses.

In our previous study that followed the glycosylation process during the chondrocyte differentiation (22), N-glycans continued to increase while GSL-glycans decreased and then leveled off as differentiation progressed; O-glycans increased temporarily and then reduced, similar to a decrease after passaging in the present study. The increase in GSLs by passage is a distinctly different phenotype from glycosylation in chondrogenic differentiation, and the glycans associated with antigenic changes in transplanted cells are likely found in GSL-glycans. The observed activation of macrophages due to alterations in the glycan profile of chondrocytes provides a possible explanation for the antigenicity and immune rejection of transplanted cells. These findings have important implications for the development of cell transplantation therapies for cartilage repair, highlighting the need to optimize cell culture conditions to minimize immune responses. Additionally, the identification of specific glycan structures involved in modulating the macrophage response provides potential targets for therapeutic intervention to improve the long-term survival and functionality of transplanted cells. Before its clinical application, the challenge of transplantation and analysis of the survival of passage-cultured cells treated with glucosylceramide synthase inhibitors remains unclear.

One limitation of this study is the use of murine models, which may not fully recapitulate the complex immune responses observed in humans. Additionally, while the GD3S-/- deficient mice provided valuable insights into the role of specific glycan structures, further studies are needed to validate these findings in human cell models and clinical settings. Moreover, the *in vitro* nature of the coculture assays may not fully reflect the *in vivo* microenvironment, highlighting the need for additional studies using animal models and clinical samples.

In conclusion, our study highlights the importance of glycan profile alterations in passaged chondrocytes in the activation of macrophages, the first line of our immune system. These findings provide novel insights into the mechanisms underlying the immune response to passaged cells, highlighting the importance of considering glycan dynamics in cell transplantation therapy and providing insights into potential strategies to increase the long-term success of such treatments. Further research is warranted to validate these findings in clinical settings and explore their translational potential for improving patient outcomes.

## Data Availability

The original contributions presented in the study are included in the article/**Supplementary Materials**. Further inquiries can be directed to the corresponding authors.
