# Supplementary material for: Associations between glycan signature alterations and the cellular antigenic properties of passaged chondrocytes
Source: Front Immunol. 2024 Nov 25;15:1475473. doi: 10.3389/fimmu.2024.1475473 (PMC11625746; doi:10.3389/fimmu.2024.1475473)
Supplement: Supplementary file 1 [file DataSheet1.docx]

Supplementary Material

**
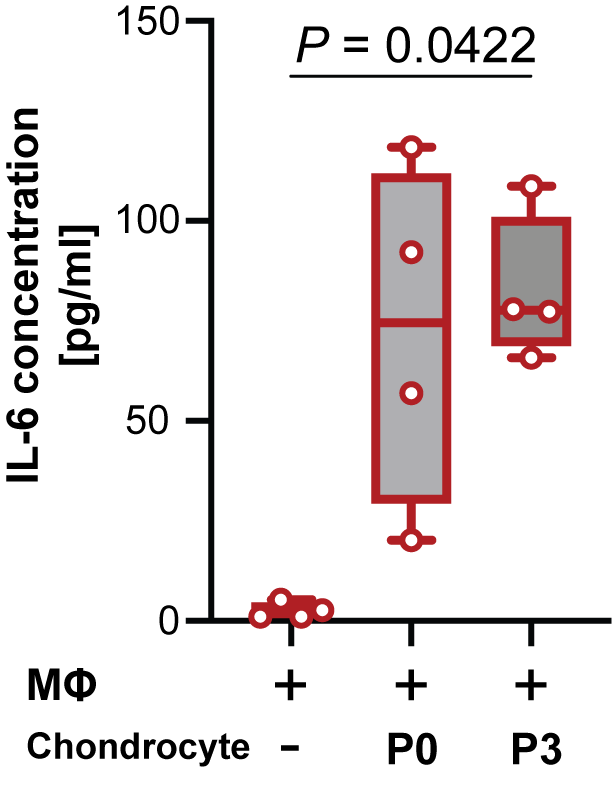
**

**Supplementary Figure S1.** Detection of IL-6 levels in the supernatant of peritoneal macrophages co-cultured with different passage numbers of chondrocytes using an indirect co-culture system (n = 4 mice). Data are mean ± s.d.; Kruskal-Wallis test. P, passage; MΦ, macrophage.


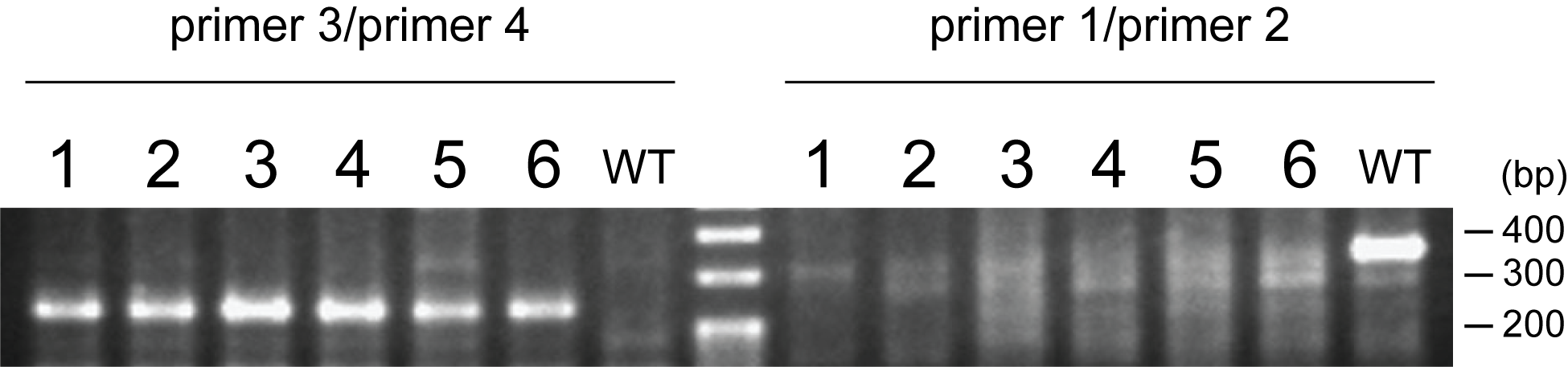


**Supplementary Figure S2.** Genotyping PCR using specific primers of 5'-AGCTCAGAGCTATGCTCAGGA-3' (primer 1), 5'-TACCACATCGAACTGGTTGAG-3' (primer 2), 5'- CAATAGATCTGACCCCTATGC-3' (primer 3), and 5'-TCGCCTTCTTGACGAGTTCTTCTGAG-3' (primer 4). For genotyping by PCR, the primers were Primers 1 and 2 detected the wild-type GM3S allele and amplified an ~400-bp fragment. Primers 3 and 4 detected the mutant allele and amplified an ~300-bp fragment. Forty-five cycles of 94°C (1 min), 60°C (1 min), and 72°C (1 min) were used for amplification. Protocol was previously reported by Yamashita et al (23).


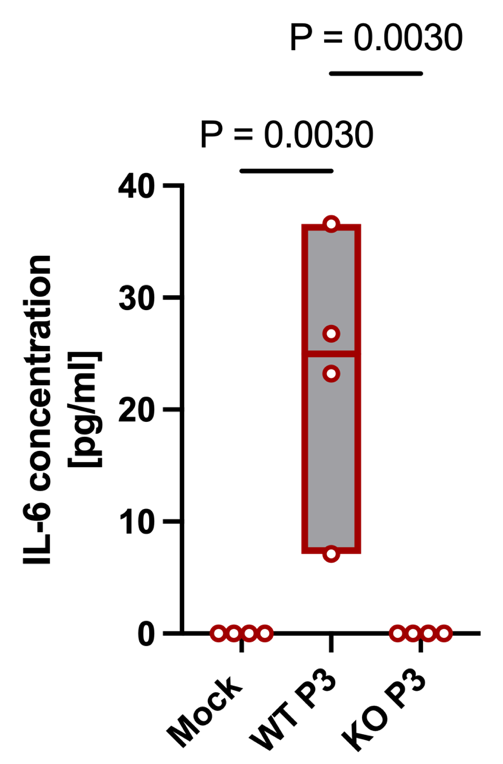


**Supplementary Figure S3.** Detection of IL-6 in the supernatant of peritoneal macrophages co-cultured with chondrocytes. Mock is a supernatant of macrophages alone. WT P3 is a supernatant of macrophages co-cultured with passaged 3 chondrocytes from WT mice. KO P3 is a supernatant of macrophages co-cultured with passaged 3 chondrocytes from *GM3S*-deficient mice. The values are shown as the mean ± standard error of the mean (mean ± SEM) of 4 measurements (n = 4). Statistical significances were determined using one-way ANOVA and were considered statistically significant when P < 0.05.

**Supplementary Table S1.** Primers used in this study.

| **Gene** |  |  |  | **Sequence** |
| --- | --- | --- | --- | --- |
| *St3gal-5* |  | Forward |  | CAAAGCAAGATGAGAAGG |
|  |  | Reverse |  | AAACTTGGGACGACATTC |
| *Il-6* |  | Forward |  | TAGTCCTTCCTACCCCAATTTCC |
|  |  | Reverse |  | TTGGTCCTTAGCCACTCCTTC |
| *Gapdh* |  | Forward |  | TGCAGCGAACTTTATTGATG |
|  |  | Reverse |  | ACTTTGTCAAGCTCATTTCC |

**Supplementary Table S2.** N-glycan list.

| No. | Putative Structure |  | | pmol/ 100 mg Proteins | | | |
| --- | --- | --- | --- | --- | --- | --- | --- |
|  |  | *m/z* | P0 | | P1 | P2 | P3 |
| N-1 | (Hex)2 (HexNAc)2 | 1178.50 | 6.15 | | 10.09 | 17.77 | 42.24 |
| N-2 | (Hex)2 (HexNAc)2 (Fuc)1 | 1324.55 | 20.24 | | 37.04 | 80.30 | 181.28 |
| N-3 | (Hex)3 (HexNAc)2 | 1340.55 | 5.28 | | 17.19 | 43.20 | 83.42 |
| N-4 | (Hex)3 (HexNAc)2 (Fuc)1 | 1486.61 | 8.34 | | 32.78 | 97.63 | 174.03 |
| N-5 | (Hex)4 (HexNAc)2 | 1502.60 | 7.34 | | 12.08 | 23.29 | 48.39 |
| N-6 | (Hex)4 (HexNAc)2 (Fuc)1 | 1648.66 | 0.78 | | 0.88 | 3.27 | 2.97 |
| N-7 | (Hex)2 + (Man)3(GlcNAc)2 | 1664.65 | 79.25 | | 65.55 | 113.15 | 209.08 |
| N-8 | (Hex)3 + (Man)3(GlcNAc)2 | 1826.71 | 218.28 | | 192.23 | 294.89 | 474.47 |
| N-9 | (Hex)4 + (Man)3(GlcNAc)2 | 1988.76 | 77.42 | | 93.81 | 164.56 | 256.74 |
| N-10 | (Hex)5 + (Man)3(GlcNAc)2 | 2150.81 | 114.08 | | 140.30 | 228.43 | 374.21 |
| N-11 | (Hex)6 + (Man)3(GlcNAc)2 | 2312.86 | 22.24 | | 75.17 | 86.97 | 143.54 |
| N-12 | (HexNAc)1 + (Man)3(GlcNAc)2 | 1543.63 | 0.00 | | 3.51 | 6.28 | 10.05 |
| N-13 | (HexNAc)1 (Fuc)1 + (Man)3(GlcNAc)2 | 1689.69 | 3.95 | | 8.56 | 12.17 | 19.68 |
| N-14 | (Hex)1 (HexNAc)1 + (Man)3(GlcNAc)2 | 1705.68 | 0.00 | | 2.25 | 2.96 | 2.40 |
| N-15 | (HexNAc)2 + (Man)3(GlcNAc)2 | 1746.71 | 0.00 | | 3.59 | 6.17 | 9.94 |
| N-16 | (Hex)2 (Fuc)1 + (Man)3(GlcNAc)2 | 1810.71 | 0.00 | | 0.81 | 3.48 | 2.67 |
| N-17 | (Hex)1 (HexNAc)1 (Fuc)1 + (Man)3(GlcNAc)2 | 1851.74 | 2.60 | | 3.07 | 3.71 | 3.09 |
| N-18 | (Hex)2 (HexNAc)1 + (Man)3(GlcNAc)2 | 1867.73 | 0.00 | | 4.15 | 5.19 | 9.82 |
| N-19 | (HexNAc)2 (Fuc)1 + (Man)3(GlcNAc)2 | 1892.76 | 23.14 | | 25.54 | 34.37 | 67.07 |
| N-20 | (Hex)1 (HexNAc)2 + (Man)3(GlcNAc)2 | 1908.76 | 0.00 | | 2.48 | 4.54 | 9.23 |
| N-21 | (HexNAc)3 + (Man)3(GlcNAc)2 | 1949.79 | 0.00 | | 0.00 | 1.02 | 0.00 |
| N-22 | (Hex)2 (HexNAc)1 (Fuc)1 + (Man)3(GlcNAc)2 | 2013.79 | 2.19 | | 3.68 | 5.00 | 3.49 |
| N-23 | (Hex)3 (HexNAc)1 + (Man)3(GlcNAc)2 | 2029.79 | 0.00 | | 2.73 | 3.28 | 3.31 |
| N-24 | (Hex)1 (HexNAc)2 (Fuc)1 + (Man)3(GlcNAc)2 | 2054.82 | 7.51 | | 7.77 | 8.82 | 17.60 |
| N-25 | (Hex)2 (HexNAc)2 + (Man)3(GlcNAc)2 | 2070.81 | 0.00 | | 2.80 | 4.51 | 4.46 |
| N-26 | (HexNAc)3 (Fuc)1 + (Man)3(GlcNAc)2 | 2095.84 | 3.19 | | 1.68 | 1.79 | 3.05 |
| N-27 | (Hex)1 (HexNAc)1 (Fuc)1 (NeuAc)1 + (Man)3(GlcNAc)2 | 2156.85 | 0.00 | | 2.35 | 1.93 | 3.45 |
| N-28 | (Hex)2 (HexNAc)2 (Fuc)1 + (Man)3(GlcNAc)2 | 2216.87 | 2.39 | | 5.33 | 9.84 | 23.66 |
| N-29 | (Hex)1 (HexNAc)3 (Fuc)1 + (Man)3(GlcNAc)2 | 2257.90 | 0.00 | | 2.11 | 0.00 | 0.00 |
| N-30 | (HexNAc)4 (Fuc)1 + (Man)3(GlcNAc)2 | 2298.92 | 0.00 | | 2.65 | 0.00 | 0.00 |
| N-31 | (Hex)1 (HexNAc)1 (Fuc)1 (NeuGc)1 + (Man)3(GlcNAc)2/(Hex)2 (HexNAc)1 (NeuAc)1 + (Man)3(GlcNAc)2 | 2172.85 | 0.00 | | 3.04 | 4.27 | 0.00 |
| N-32 | (Hex)2 (HexNAc)1 (Fuc)1 (NeuGc)1 + (Man)3(GlcNAc)2 | 2334.90 | 0.00 | | 1.10 | 1.36 | 0.00 |
| N-33 | (Hex)1 (HexNAc)2 (Fuc)1 (NeuGc)1 + (Man)3(GlcNAc)2/(Hex)2 (HexNAc)2 (NeuAc)1 + (Man)3(GlcNAc)2 | 2375.93 | 1.62 | | 3.45 | 3.79 | 3.24 |
| N-34 | (Hex)1 (HexNAc)2 (Fuc)2 (NeuGc)1 + (Man)3(GlcNAc)2/(Hex)2 (HexNAc)2 (Fuc)1 (NeuAc)1 + (Man)3(GlcNAc)2 | 2521.99 | 0.00 | | 2.19 | 4.34 | 4.68 |
| N-35 | (Hex)2 (HexNAc)2 (NeuAc)2 + (Man)3(GlcNAc)2 | 2681.04 | 4.70 | | 0.00 | 0.00 | 0.00 |
| N-36 | (Hex)2 (HexNAc)2 (Fuc)2 (NeuGc)1 + (Man)3(GlcNAc)2/(Hex)3 (HexNAc)2 (Fuc)1 (NeuAc)1 + (Man)3(GlcNAc)2 | 2684.04 | 0.00 | | 3.14 | 5.73 | 10.17 |
| Total |  |  | 610.68 | | 775.11 | 1288.01 | 2201.44 |

**Supplementary Table S3.** O-glycan list.

| No. | Putative Structure | m/z | pmol/ 100 mg Proteins | | | |
| --- | --- | --- | --- | --- | --- | --- |
|  |  |  | P0 | P1 | P2 | P3 |
| O-1 | (Hex)1 | 510.42 | 493.35 | 85.91 | 149.94 | 158.78 |
| O-2 | (HexNAc)1 | 551.47 | 60.66 | 100.40 | 188.65 | 165.08 |
| O-3 | (Hex)1 (HexNAc)1 | 713.71 | 5.12 | 8.31 | 17.91 | 15.02 |
| O-4 | (HexNAc)2 | 754.76 | 0.00 | 0.75 | 1.36 | 0.92 |
| O-5 | (HexNAc)1 (NeuAc)1 | 842.84 | 0.91 | 1.51 | 2.32 | 3.03 |
| O-6 | (Hex)1 (HexNAc)2 | 916.77 | 0.43 | 0.48 | 0.67 | 0.62 |
| O-7 | (Hex)1 (HexNAc)1 (NeuAc)1 | 1005.06 | 12.15 | 27.66 | 46.86 | 43.00 |
| O-8 | (Hex)2 (HexNAc)2 | 1079.04 | 0.00 | 0.51 | 1.28 | 1.07 |
| O-9 | (Hex)1 (HexNAc)2 (NeuAc)1 | 1208.19 | 0.00 | 2.27 | 2.44 | 2.32 |
| O-10 | (Hex)1 (HexNAc)1 (NeuAc)2 | 1296.35 | 9.00 | 6.03 | 10.01 | 10.41 |
| O-11 | (Hex)2 (HexNAc)2 (NeuAc)1 | 1370.24 | 0.33 | 0.64 | 1.34 | 0.82 |
| O-12 | (Hex)2 (HexNAc)2 (NeuAc)2 | 1661.42 | 0.41 | 0.77 | 1.41 | 0.88 |
| Total |  |  | 582.35 | 235.23 | 424.18 | 401.96 |

**Supplementary Table S4.** GSL-glycan list.

| No. | Putative Structure | *m/z* | pmol/ 100 mg Proteins | | | |
| --- | --- | --- | --- | --- | --- | --- |
|  |  |  | P0 | P1 | P2 | P3 |
| GSL-1 | (Hex)2 | 772.34 | 45.62 | 65.63 | 63.33 | 32.60 |
| GSL-2 | (Hex)3 | 934.39 | 36.25 | 23.02 | 20.61 | 17.28 |
| GSL-3 | (Hex)2(HexNAc)1 | 975.42 | 22.57 | 31.43 | 24.46 | 13.16 |
| GSL-4 | (Hex)2(Neu5Ac)1-lactone1 | 1045.43 | 0.00 | 33.04 | 45.27 | 33.79 |
|  | (Hex)2(Neu5Ac)1 | 1077.45 | 46.13 | 506.73 | 780.29 | 612.14 |
| GSL-5 | (Hex)2(Neu5Gc)1 | 1093.45 | 0.00 | 9.03 | 13.74 | 5.02 |
| GSL-6 | (Hex)4 | 1096.44 | 48.39 | 10.82 | 13.87 | 10.87 |
| GSL-7 | (Hex)3(HexNAc)1 | 1137.47 | 204.56 | 312.31 | 523.87 | 419.66 |
| GSL-8 | (Hex)2(HexNAc)2 | 1178.50 | 5.61 | 33.62 | 61.36 | 47.44 |
| GSL-9 | (Hex)5 | 1258.49 | 5.53 | 0.00 | 0.00 | 0.00 |
| GSL-10 | (Hex)2(HexNAc)1(Neu5Ac)1 | 1280.53 | 0.00 | 14.37 | 23.29 | 16.94 |
| GSL-11 | (Hex)4(HexNAc)1 | 1299.52 | 21.82 | 36.87 | 22.25 | 11.58 |
| GSL-12 | (Hex)3(HexNAc)2 | 1340.55 | 7.12 | 10.32 | 12.04 | 12.22 |
| GSL-13 | (Hex)2(Neu5Ac)2 -lactone1 | 1350.54 | 232.38 | 9.40 | 2.21 | 0.00 |
|  | (Hex)2(Neu5Ac)2 | 1382.57 | 7.07 | 0.00 | 0.00 | 0.00 |
| GSL-14 | (Hex)6 | 1420.55 | 3.07 | 0.00 | 0.00 | 0.00 |
| GSL-15 | (Hex)3(HexNAc)1(Neu5Ac)1 | 1442.58 | 7.86 | 66.79 | 166.99 | 288.91 |
| GSL-16 | (Hex)5(HexNAc)1 | 1461.57 | 32.14 | 18.64 | 16.29 | 9.38 |
| GSL-17 | (Hex)4(HexNAc)2 | 1502.60 | 0.00 | 6.35 | 6.41 | 6.70 |
| GSL-18 | (Hex)3(HexNAc)3 | 1543.63 | 0.00 | 4.57 | 5.14 | 2.61 |
| GSL-19 | (Hex)6(HexNAc)1 | 1623.63 | 5.67 | 0.00 | 1.82 | 0.00 |
| GSL-20 | (Hex)3(HexNAc)1(Neu5Ac)2-lactone1 | 1715.67 | 1.75 | 16.15 | 74.92 | 184.48 |
|  | (Hex)3(HexNAc)1(Neu5Ac)2 | 1747.70 | 4.21 | 207.60 | 1115.17 | 2691.26 |
| GSL-21 | (Hex)3(HexNAc)1(Neu5Ac)1(Neu5Gc)1 | 1763.69 | 0.00 | 0.74 | 14.01 | 40.04 |
| GSL-22 | (Hex)3(HexNAc)3(NeuAc)1 | 1848.74 | 0.00 | 13.16 | 12.63 | 16.75 |
| GSL-23 | (Hex)3(HexNAc)2(NeuAc)2 | 1950.78 | 0.00 | 7.87 | 8.36 | 32.91 |
| Total |  |  | 737.77 | 1438.45 | 3028.34 | 4505.76 |
